# Understanding the Complex Patterns Observed during Hepatitis B Virus Therapy

**DOI:** 10.3390/v9050117

**Published:** 2017-05-19

**Authors:** Andrea Carracedo Rodriguez, Matthias Chung, Stanca M. Ciupe

**Affiliations:** Department of Mathematics, Virginia Tech, 460 McBryde Hall, Blacksburg, VA 24060, USA; crandrea@vt.edu (A.C.R.); mcchung@vt.edu (M.C.)

**Keywords:** virus kinetics, hepatitis B virus (HBV), drug therapy, Monte Carlo

## Abstract

Data from human clinical trials have shown that the hepatitis B virus (HBV) follows complex profiles, such as bi-phasic, tri-phasic, stepwise decay and rebound. We utilized a deterministic model of HBV kinetics following antiviral therapy to uncover the mechanistic interactions behind HBV dynamics. Analytical investigation of the model was used to separate the parameter space describing virus decay and rebound. Monte Carlo sampling of the parameter space was used to determine the virological, pharmacological and immunological factors that separate the bi-phasic and tri-phasic virus profiles. We found that the level of liver infection at the start of therapy best separates the decay patterns. Moreover, drug efficacy, ratio between division of uninfected and infected cells, and the strength of cytotoxic immune response are important in assessing the amount of liver damage experienced over time and in quantifying the duration of therapy leading to virus resolution in each of the observed profiles.

## 1. Introduction

Hepatitis B virus (HBV) infection affects 250–350 million people worldwide [1]. Universal HBV vaccination has reduced the acquisition, spread, and prevalence of HBV infection [2]. Despite these advances in HBV control, chronic infections are the main reason for the development of liver cirrhosis and cancer [3]. According to reports by the World Health Organization, mortality related to liver diseases within hepatitis B chronically-infected individuals reaches 25–30%, and improved treatments are desirable [4].

Two types of drug therapies have been approved for treating HBV chronic disease: interferon (standard and PEGylated) and nucleos(t)ide analogs [5]. These medications suppress HBV replication and liver inflammation but do not lead to a cure. The interferon-α treatments modulate immune responses that may lower viral levels. It is given for a finite time (usually 12 months) due to its toxic side effects [5,6]. The nucleo(t)side analogues are administered for many years (and sometimes for life) and are responsible for viral suppression. However, life-long therapy is difficult due to costs, compliance and most importantly the development of antiviral drug resistance [7]. Combination therapies have not shown an increased effect on treatment response, however they have led to a reduced rate of drug resistance [8]. The limitations of these therapies hinder establishing universal guidelines for treatment start, duration, type, or combinations of drugs to use [9] and how to define the success of therapy (virologically, serologically, and/or immunologically) [1,9].

Insight into the trade-off between the duration and strength of therapy, the role of immune responses, and the therapy outcome can be provided through mathematical modeling [10,11,12]. The mechanistic interactions that cause the complex hepatitis B virus patterns have been captured by a model developed by Dahari et al. [12]. These observed patterns during drug trials include bi-phasic, tri-phasic, and stepwise hepatitis B virus decays, but also initial decay followed by a flat stage and viral rebound. The study predicts that the observed virus patterns depend mainly on four biological features: ratio of uninfected to infected cells at the start of therapy, total number of liver cells at start of therapy, patient-specific critical drug effectiveness, and overall drug effectiveness [12]. In our work, we will expand these results to account for the connection between the observed viral decay patterns and parameters in the model accounting for liver cell proliferation, immune responses and drug efficacy. We utilize statistical and numerical methods to determine the parameter regions corresponding to observed viral patterns. Particularly, we investigate the connection between viral dynamics, duration of drug therapy, viral clearance, liver damage and the level of infection at the start of the treatment. We found that the level of liver infection at the start of therapy best separates the decay patterns. Moreover, drug efficacy, ratio between division of uninfected and infected cells, and the strength of cytotoxic immune response are important in assessing the amount of liver damage experienced over time and in quantifying the duration of therapy leading to virus resolution in each of the observed profiles.

The paper is structured as follows. In Section 2, we lay out the model and the analytical and statistical methods utilized in its investigation. In Section 3, we present the connection between the type of viral decay and (1) model parameters; (2) the duration of treatment and (3) liver toxicity and the size of infection at the start of therapy. In Section 4, we end with a discussion.

## 2. Materials and Methods

### 2.1. Mathematical Model

The goal of our study is to investigate the virological and immunological factors that can explain the complex hepatitis B virus dynamics seen in drug therapies. To address this, we analytically and numerically investigate a modified version of the mathematical model from Dahari et al. [12]. The model considers the interaction between uninfected hepatocytes *T*, the productively infected hepatocytes *I*, and the hepatitis B virus *V*. Briefly, both uninfected and infected hepatocytes are maintained by homeostatic proliferation described by a logistic equation, with carrying capacity *K* and maximal per capita division rates $r_{T}$ and $r_{I}$, respectively. It assumes that an infected cell produces two infected offspring. This assumption is an oversimplification and may not always be true, as virus proteins may be lost during replication, leading to production of uninfected offspring [13,14]. HBV infection occurs at rate β. Infected cells are lost at rate δ, which accounts for T cell-mediated cytotoxic killing. Infected cells produce *p* virions and virus is cleared at rate *c*. The combination therapy (with interferon-α and nucleos(t)ide drugs) lowers both virus infectivity and virus production by 1−η and 1−ϵ, respectively; where 0≤η,ϵ≤1 are drug efficacy. The model describing these interactions is given by:

(1) $$\begin{array}{c} \begin{array}{cc} \frac{d\;T}{d\;t} & =r_{T}T\left(1-\frac{T+I}{K}\right)-\beta \left(1-\eta \right)VT,\\ \frac{d\;I}{d\;t} & =r_{I}I\left(1-\frac{T+I}{K}\right)+\beta \left(1-\eta \right)VT-\delta I,\\ \frac{d\;V}{d\;t} & =(1-\epsilon )pI-cV. \end{array} \end{array}$$

**Figure 1:**
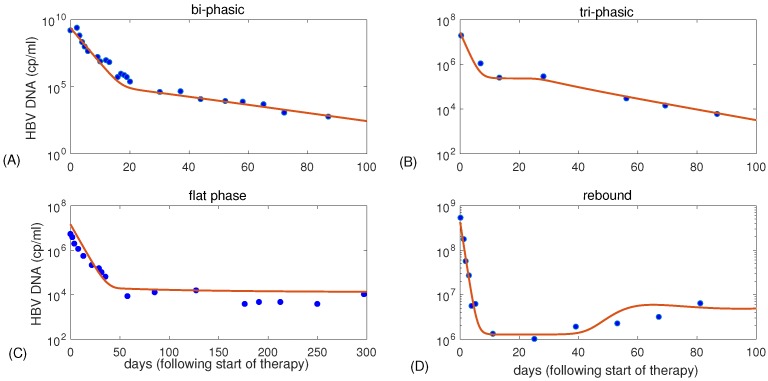
Profiles of V(t) per ml given by model (1) and parameters in Table (1). Data was digitized from (**A**) Lewin et al. [10]; (**B**) Tsiang et al. [11]; (**C**) Colombatto et al. [18]; and (**D**) Lewin et al. [10]. HBV: hepatitis B virus.

We assume that a patient is chronically infected at the start of the treatment. We model that by assuming that system (1) in the absence of drug therapy (η=ϵ=0) has reached the positive equilibrium solution $E=(\bar{T},\bar{I},\bar{V})$:

(2) $$\begin{array}{cc} \bar{T} & =\frac{c\delta (R_{0}\left(\delta -r_{I}\right)+r_{T})}{\beta p(\delta R_{0}+r_{T}-r_{I})},\\ \bar{I} & =\frac{c\delta r_{T}\left(R_{0}-1\right)}{\beta p(\delta R_{0}+r_{T}-r_{I})},\\ \bar{V} & =\frac{\delta r_{T}\left(R_{0}-1\right)}{\beta (\delta R_{0}+r_{T}-r_{I})}, \end{array}$$

where,

(3) $$R_{0}=\frac{pK\beta }{c\delta }$$

is the basic reproduction number. The system reaches the equilibrium point $E=(\bar{T},\bar{I},\bar{V})$ when,

(4) $$1<R_{0}<\frac{r_{T}}{r_{I}-\delta },$$

and $r_{I}>\delta$, i.e., *E* is locally asymptotically stable (see supplementary material for details). Note that system (1) has another chronic equilibrium:

$$E^{tot.liv}=\left(0,\frac{K(r_{I}-\delta )}{r_{I}},\frac{pK(r_{I}-\delta )}{cr_{I}}\right),$$

corresponding to the entire liver being infected. This equilibrium exists when $r_{I}>\delta$, and is stable when,

(5) $$R_{0}\frac{r_{I}-\delta }{r_{T}}>1,$$

(see supplementary material for details). While having the entire liver infected during acute infections is biologically feasible [15], we chose to not start drug therapy at this equilibrium. The reason is purely technical, and it is due to the fact that, in the context of the ordinary differential equation (ODE) model (1), the uninfected hepatocyte population will never recover (after treatment is initiated) when we start with a zero initial condition. Data from drug clinical trials shows that in most patients HBV DNA removal follows a bi-phasic pattern [11,16,17]. Other viral patterns, such as tri-phasic decay, rebound, and decay to a positive flat phase have also been observed [12,18]. Model (1) is able to capture all of these V(t) dynamics by varying model’s parameters (Figure 1A–D, for parameters in Table 1).

We are interested in determining (1) under what conditions model (1) predicts virus removal for non-negative drug rates 0<ϵ,η≤1; and (2) the patterns of virus decay. Analytically, we can show V(t) is removed in the long run when:

(6) $$R=\left(1-\eta \right)\left(1-\epsilon \right)R_{0}<1,$$

and persists when R>1, i.e., the equilibrium solution $E_{0}=\left(K,0,0\right)$ is locally asymptotically stable (unstable, respectively) (see supplementary material for details). This analytical result (6), however, cannot differentiate between the different patterns of long run virus decay.

In this study, we define and characterize the different types of long run viral decay given by model (1) using numerical and statistical techniques. We will define that V(t) decays in a bi-phasic manner if the temporal variable V(t) is convex, and in a tri-phasic manner otherwise (Figure 1, subplots A and B). As we observe in Figure 1B, the tri-phasic virus decay pattern is interrupted by a flat (not necessarily zero sloped) phase. This flat phase distinguishes the bi-phasic and tri-phasic dynamics. Assume the flat phase starts at $t_{1}$ and ends at $t_{2}$, then the length of the flat phase is $L=t_{2}-t_{1}$, while L=0 for bi-phasic virus dynamics V(t). Note that we define tri-phasic dynamics based on V(t) being concave and that a convex function that has three slopes of decay (as in [19]) will be called bi-phasic in our study.

### 2.2. Monte Carlo Sampling

Our goal is to determine the parameter regions for which the solution of V(t) given by model (1) follows bi-phasic or tri-phasic dynamics. Since an analytical investigation of the virus dynamics is unavailable, we aim for a statistical analysis.

Note that all parameters in model (1) have clear biological interpretations. The values and ranges are taken from previous modeling studies [10,11,12,20,21,22]. Throughout this paper we consider the parameters $\theta_{f}=\left\{\beta ,K,p,c,\eta \right\}$ to be kept as known values (Table 2). The remaining parameters, $\theta_{v}=\{r_{T},r_{I},\delta$, ϵ}, are assumed to have known ranges (Table 2). Although these ranges are considered feasible, little is known about the correlation between a set of parameter values and the corresponding dynamics of solutions T(t),I(t), and V(t). For instance, although the parameters $r_{T}=2.5$ $d^{-1}$, $r_{I}=2.5$ $d^{-1}$, δ=0.08 $d^{-1}$ and ϵ=0.75 are feasible choices according to Table 2, they lead to infeasible initial conditions for model (1). Indeed, the initial condition (2) results in negative concentration, $\bar{T}\approx -1.1489\;\times \;10^{8}$ cells per mL.

In this study we numerically investigate the dynamics of model (1) with positive initial condition $(\bar{T},\bar{I},$$\bar{V})$ given by (2) under the following conditions:

MC.1 The initial condition (2) exists and is stable, i.e., $r_{I}>\delta$ and $1<R_{0}<\frac{r_{T}}{r_{I}-\delta }$.
MC.2 The components of initial condition (2) are bound by $0<\bar{T}<K$ cells per ml, $0<\bar{I}<K$ cells per mL, and $0<\bar{V}<1.9\times 10^{10}$ virus per ml.
MC.3 The drug-dependent virus asymptotic removal condition (6) holds, i.e., 0<R<1. This condition guaranties that we are only examining drug therapy that is efficient in removing the hepatitis B virus.
MC.4 The total hepatocyte population is bound as follows 0.2K≤T(t)+I(t)≤K cells per mL, for all *t*. This condition guarantees that the total liver cell population never decays below 20% of the healthy hepatocyte concentration *K* [23].

In order to find model parameters which lead to bi-phasic and tri-phasic V(t) dynamics, we sample the hypercube determined by the parameter space of four parameters $\theta_{v}=\{r_{T},r_{I},\delta$, ϵ} with ranges shown in Table 2. We are using uniform Monte Carlo sampling and reject parameter samples which are considered infeasible, as described above. This method is called rejection sampling or the accept–reject algorithm and is well established in statistics [24].

Using this statistical method, we aim to determine which parameter regions are predominantly leading to bi-phasic and tri-phasic V(t) dynamics. We first investigate the entire parameter space, followed by constrains to regions where (1) the ratio of uninfected to infected cells division rate is fixed; (2) the death rate of infected cells is fixed; and (3) drug therapy has high efficacy. These restrictions will help distinguish between the effects that liver reconstitution, the immune cell killing rate, and the drug efficacy have on the pattern of long run virus decay. Lastly, we determine the role of liver turnover, net liver mass gain, speed of virus resolution and the level of liver infection prior to therapy in determining the viral decay pattern.

## 3. Results

### 3.1. Parameter Regions for Bi-Phasic and Tri-Phasic Virus Dynamics

Initially, we investigate the entire parameter space by randomly generating $10^{8}$ parameter sets for the vector $\theta_{v}$, with the ranges for each component kept between the boundaries given in Table 2. For each sample, we solve system (1) subject to initial conditions (2) and keep only the samples that satisfy conditions MC.1–MC.4 in Section 2.2. We present all results in terms of the ratio $r_{T}/r_{I}$, instead of the individual $r_{T}$ and $r_{I}$ values, since the V(t) patters correlate with the ratio of uninfected to infected proliferation rates, rather than the individual proliferation rates.

**Figure 2:**
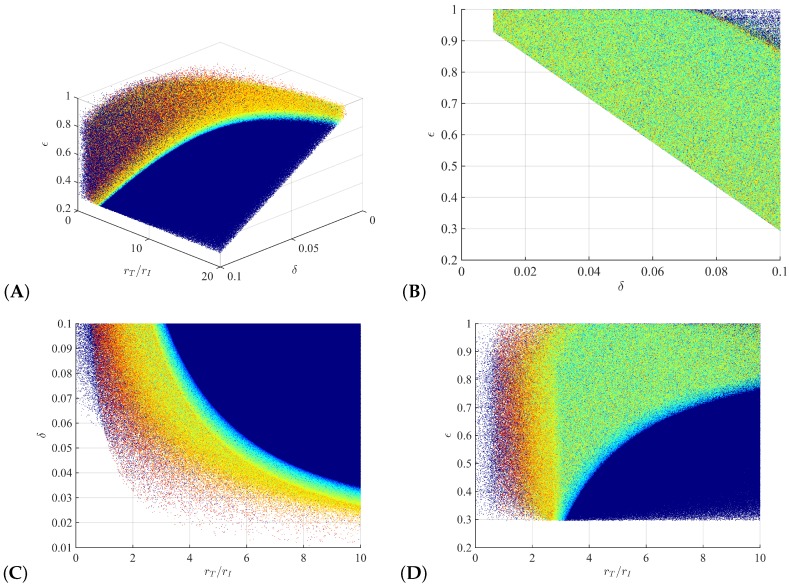
(**A**) Samples for bi-phasic (dark blue dots) and tri-phasic (red to light blue dots) V(t) patterns in the three-dimensional $r_{T}/r_{I}$-δ-ϵ space; (**B**) Projection of the samples in (**A**) onto the δ-ϵ space; (**C**) Projection of the samples in (**A**) onto the $r_{T}/r_{I}$-δ space; (**D**) Projection of the samples in (**A**) onto the $r_{T}/r_{I}$-ϵ space.

**Figure 3:**
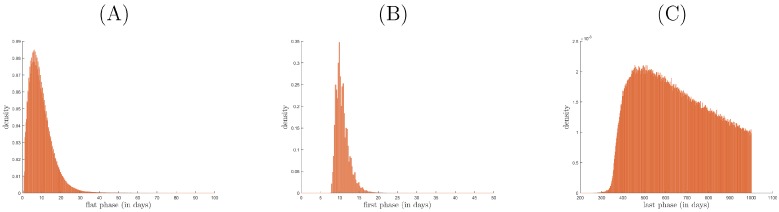
Distribution of the tri-phasic V(t) samples versus the length in days of: (**A**) flat phase; (**B**) first slope; (**C**) last slope.

We obtain $∼8.2\cdot 10^{7}$ and $∼1.8\cdot 10^{7}$ (82% vs. 18%) parameter samples for bi-phasic and tri-phasic V(t) dynamics, respectively (Figure 2A, dark blue versus red-to-light blue dots, respectively). To distinguish further between the different types of tri-phasic decays, we color code the tri-phasic samples, such that the samples with a longer flat phase *L* have a darker red shade. Moreover, we compare the length of the flat phase *L* and its frequency among the samples, and find it to average at 10 days, with a range of 1 to 30 days (see Figure 3A). Moreover, the first phase decay has an average of 10 days, while the last phase has an average of 500 days (see Figure 3B,C).

We observe that the parameter space can be separated into two subregions: a parameter subregion in which all samples correspond to bi-phasic V(t) dynamics (right hand side of Figure 2A) and a parameter subregion that cannot distinguish between bi-phasic and tri-phasic V(t) dynamics (left hand side of Figure 2A).

Projections of the parameter space into 2D graphs show: (1) either a large ratio between uninfected and infected cell division rate $r_{T}/r_{I}$, a high cytotoxic killing rate δ, or a high drug efficacy are needed for V(t) long run removal (see Figure 2B,C); (2) high values for both the $r_{T}/r_{I}$ ratio and the cytotoxic killing rate δ guarantee bi-phasic V(t) decay patterns (see Figure 2C); (3) 30% or higher drug efficacy rate is needed for V(t) long run removal (see Figure 2B,D); and (4) both bi-phasic and tri-phasic patterns can be seen for intermediate drug efficacy 0≤ϵ≤0.8 (see Figure 2D).

**Figure 4:**
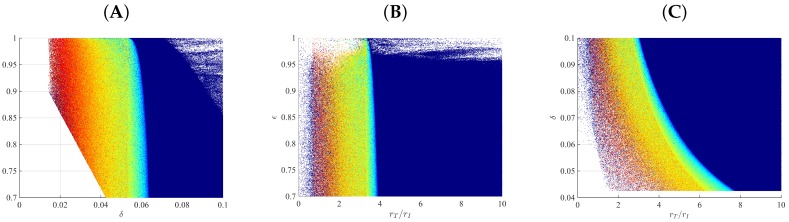
Samples for bi-phasic (dark blue dots) and tri-phasic (red to light blue dots) V(t) dynamics for: (**A**) fixed $r_{T}/r_{I}=5$; (**B**) fixed δ=0.08; (**C**) fixed ϵ=0.7. The other parameters are as in Table 2.

To better determine the relative effects of $r_{T}/r_{I}$, δ and ϵ, we generate new samples for the following three subcases (S1, S2, and S3). In the subcase S1, we assume that δ is in the range given in Table 2, there is at least 70% drug efficacy (ϵ≥0.7), and the ratio between uninfected to infected cell division rate $r_{T}/r_{I}$ is fixed. If we assume that $r_{T}/r_{I}=5$, corresponding to the situation where the main goal is to maintain a high uninfected liver population, then of the $10^{8}$ samples considered, $∼7.5\times 10^{7}$ and $∼2.5\times 10^{7}$ samples (75% vs. 25%) correspond to bi-phasic and tri-phasic V(t) dynamics, respectively. Samples with small values for both δ and ϵ (δ<0.03 $d^{-1}$, and ϵ<0.8) are rejected by our method because they violate at least one of the conditions MC.1–MC.4 in Section 2.2. For the accepted samples, we find that the pattern of V(t) long run decay is independent of the drug efficacy ϵ, for a fixed $r_{T}/r_{I}$ value. High cytotoxic killing rates δ>0.065 $d^{-1}$ correspond to bi-phasic V(t) patterns (see Figure 4A), while low cytotoxic killing rates δ<0.055 $d^{-1}$ correspond to a mixture of bi-phasic and tri-phasic V(t) patterns, regardless of the efficacy of antiviral therapy ϵ (see Figure 4A). If we decrease the $r_{T}/r_{I}$ ratio to 2.5 and 1, respectively, then high cytotoxic killing rates are needed for long run V(t) removal. Moreover, it gets impossible to separate the parameter space corresponding to bi-phasic and tri-phasic regions (see Figure S1A,B).

In the subcase S2, we assumed that the uninfected and infected cell division rates $r_{T}$ and $r_{I}$ are in the ranges given in Table 2, the drug efficacy is higher than 70% (0.7≤ϵ≤1), and cytotoxic killing rate δ is fixed. If we consider δ=0.08 $d^{-1}$, corresponding to infected cells lifespan of 12.5 days (eight times smaller than that of uninfected cells) then, of the $10^{8}$ parameter samples generated, $∼8.5\times 10^{7}$ and ∼$1.5\times 10^{7}$ (85% vs. 15%) have bi-phasic and tri-phasic V(t) patterns, respectively. We observe bi-phasic V(t) patterns when the ratio between uninfected and infected hepatocyte division rate is bigger than 4, and a mixture of bi-phasic and tri-phasic patterns otherwise (see Figure 4B). This implies that, for a fixed δ value, we may find a flat phase during therapy if the proliferation of the uninfected target cells does not, at least, quadruple the proliferation of infected cells. If we decrease the cytotoxic killing rate to δ=0.01 corresponding to the infected hepatocytes lifespan of 100 days, then higher than 93% drug efficacy is needed for long run V(t) removal (see Figure S1E). Moreover a large $r_{T}/r_{I}$ ratio (larger than 40) is needed for bi-phasic V(t) patterns (see Figure S1E).

Lastly, in case S3, we keep the the uninfected and infected cell division rates $r_{T}$ and $r_{I}$ in the ranges given in Table 2 (as before), fix drug therapy ϵ and vary the cytotoxic killing rate δ as in Table 2, which corresponds to death rates of infected cells ranging between non-infection natural death rate and a 10-times faster death rate, due to immune mediated killing. If ϵ=0.7, then of the $10^{8}$ parameter samples, we obtain $∼8.2\times 10^{7}$ and $∼1.8\times 10^{7}$ (82% vs. 18%) bi-phasic and tri-phasic V(t) patterns. Samples for which the killing rate’s half-life was smaller than 16.3 days (δ<0.0425 $d^{-1}$) were rejected regardless of the size of the $r_{T}/r_{I}$ ratio, implying that immune mediated killing is needed for virus long run removal as observed in other studies [12]. For this case, the parameter region for a bi-phasic V(t) pattern requires a combination of high $r_{T}/r_{I}$ and δ values. Conversely, low $r_{T}/r_{I}$ and δ values give rise, once again, to a mixture of bi-phasic and tri-phasic V(t) patterns (see Figure 4C). When we increase drug efficacy to ϵ=0.9 and ϵ=0.99, respectively, then we obtain virus long run decay for all the δ values considered (see Figure S1C,D).

In all considered cases, we were unable to properly distinguish the parameter regions that completely separate the bi-phasic and tri-phasic V(t) patterns. We next look into whether biologically relevant factors, such as the amount of liver loss, the net liver gain, the time to virus removal, and the relative size of the uninfected and infected hepatocyte populations at the beginning of the treatment can further explain the V(t) decay patterns. We define virus clearance time, the time $t_{\mathrm{ext}}$ at which a solution V(t) reaches one virion ($V\left(t\right)=V_{\mathrm{ext}}=3\times 10^{-4}$ per mL, for virus concentration distributing through 3 L of blood). We call $V_{\mathrm{ext}}$ the virus clearance level.

### 3.2. Relationship between V(t) Patterns and the Duration of Treatment

Since we could not properly classify the samples, giving bi-phasic and tri-phasic V(t) dynamics for all parameter samples, we investigate whether there is a connection between the observed pattern and the duration of the treatment till viral clearance. Intuitively, a bi-phasic V(t) should correlate with successful treatment, since there is no virus stagnation as in the flat phase of a tri-phasic sample. We define successful and partially successful treatments; therapies that lead to HBV DNA clearance after one and two years of therapy.

**Figure 5:**
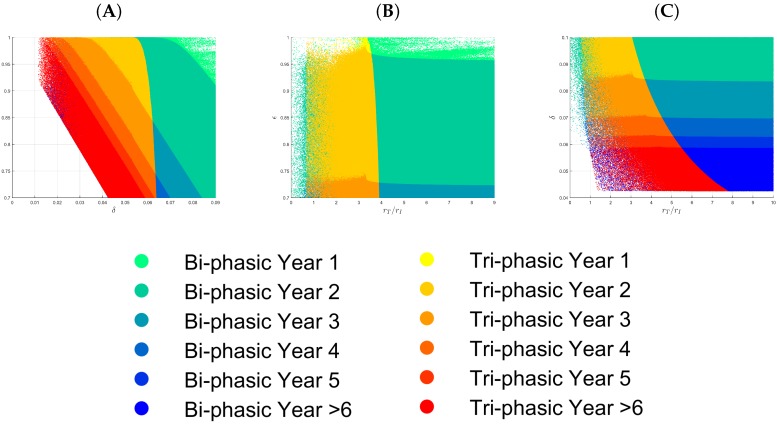
Division of the samples for bi-phasic and tri-phasic virus pattern in Figure 4 based on the number of years to virus clearance for: (**A**) fixed $r_{T}/r_{I}=5$; (**B**) fixed δ=0.08; (**C**) fixed ϵ=0.7. The other parameters are as in Table 2.

To test this intuition, we grouped the samples obtained in the three subcases S1–S3 (described in Section 3.1) by the number of years of therapy needed such that $V\left(t\right)\leq V_{\mathrm{ext}}$ (see Figure 5 and Figure S2). We have considered six groups, corresponding to viral removal happening during the first to five and longer than five years of viral therapy. Our simulations show that while for the most samples the time to viral removal is similar regardless of V(t) pattern of decay (see Figure 5A), some bi-phasic samples in the overlapping region (left side of the parameter space) have a shorter time to viral removal compared to the tri-phasic samples in the same region (not shown). These results suggest that, in general, bi-phasic V(t) patterns correlate to faster virus clearance compared to the tri-phasic V(t) patterns.

We further look into the relationship between the parameter space, the year of virus clearance following the start of therapy, and the type of V(t) decay pattern. We find that, for fixed δ and ϵ, the time of viral removal does not depend on the value of the ratio $r_{T}/r_{I}$ (see Figure 5B,C). As expected, drug efficacy is important for fast viral removal. When the drug efficacy is smaller than 85% (ϵ<0.85) virus removal requires therapy lasting longer than two years (see Figure 5A,B). Interestingly, the exact duration of treatment is influenced by the strength of the patient’s cytotoxic immune response, with high δ correlating with faster V(t) removal (see Figure 5A,C). As expected, higher drug efficacy ϵ=0.9 and ϵ=0.99 allow for better therapy prognosis (see Figure S2A,B). Indeed, virus clearance in the first year occurs for all samples with δ>0.7 and fixed ϵ=0.99, regardless of the $r_{T}/r_{I}$ ratio (see Figure S2A).

Once again, bi-phasic and tri-phasic V(t) patterns cannot be separated based on the duration of treatment till viral removal. We next looked at the amount of liver turnover and net liver gain predicted by the model for each sample considered.

### 3.3. Liver Toxicity and the Role of Initial Conditions

During drug therapy, the immune response is active in removing infected cells. While the hepatitis B virus does not kill liver cells directly, during chronic infections patients do experience liver loss due to cytotoxic immune responses [25]. Liver integrity is maintained through its ability to regenerate and compensate for liver killing through increased hepatocyte proliferation [26]. If at any moment the liver is reduced below 20%, the patient dies [23]. To account for this fact in model (1), we only accepted the samples for which the total amount of liver cells at each time point during treatment exceeds 20% of the total liver concentration, i.e., T(t)+I(t)≥0.2K, for all *t*.

We define the hepatocyte turnover(τ) to be the percentage liver loss due to immune response killing over the first τ days. Analytically that is:

(7) $$\mathrm{Hepatocyte}\;\mathrm{turnover}\left(\tau \right)=\int_{0}^{\tau }\delta \frac{I(t)}{K}d\;t.$$

The liver regenerates over the first τ days due to division of both uninfected and infected hepatocytes. The total liver production (τ) is given by:

(8) $$\mathrm{Total}\;\mathrm{liver}\;\mathrm{production}\left(\tau \right)=\int_{0}^{\tau }\left(r_{T}\frac{T(t)}{K}+r_{I}\frac{I(t)}{K}\right)\left(1-\frac{T(t)+I(t)}{K}\right)d\;t.$$

Finally, the net liver gain (τ) is given by difference between cell production and cell killing:

(9) $$\mathrm{Net}\;\mathrm{liver}\;\mathrm{gain}\left(\tau \right)=\int_{0}^{\tau }\left\{\left(r_{T}\frac{T(t)}{K}+r_{I}\frac{I(t)}{K}\right)\left(1-\frac{T(t)+I(t)}{K}\right)-\delta \frac{I(t)}{K}\right\}d\;t.$$

We determine the amount of liver turnover and net liver gain (in percentages) for the first τ=100 days after the start of therapy. We created data for a fourth subcase S4, where we fixed $r_{T}/r_{I}=5$, ϵ=0.7 and varied δ in the ranges given in Table 2. This means we have a fixed drug efficacy of 70% (ϵ=0.7), a 5-times better chance of an uninfected cell to divide compared to an uninfected hepatocyte ($r_{T}/r_{I}=5$) and normal to 10-times stronger than normal cytotoxic killing rate, 0.01≤δ≤0.1 $d^{-1}$. For these assumptions we randomly generate $10^{8}$ parameter samples, solve model (1) subject to initial conditions (2), and separate the results into bi-phasic and tri-phasic V(t) patterns. We find that ∼$7.7\times 10^{7}$ and $2.3\times 10^{7}$ (77% vs. 23%) samples corresponded to the bi-phasic and tri-phasic case, respectively. For each sample, we determine the relationship between the amount of liver turnover given by (7), the net liver gain given by (9), and the V(t) decay pattern (under the fixed $r_{T}/r_{I}$ and fixed ϵ assumptions).

**Figure 6:**
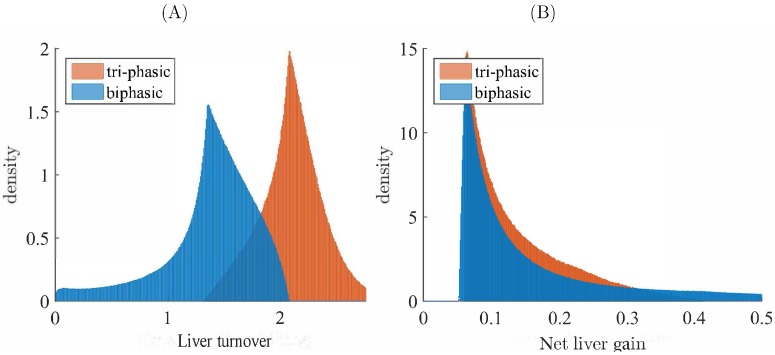
Density of bi-phasic (blue) and tri-phasic (pink) V(t) samples versus: (**A**) Liver turnover; (**B**) Net liver gain, for ϵ=0.7, $r_{T}/r_{I}=5$, 0.01≤δ≤0.1 $d^{-1}$, and τ=100 days.

In subcase S4, we find that the amount of liver turnover due to immune response-induced killing is higher for tri-phasic than for bi-phasic V(t) patterns (see Figure 6A). In particular, model (1) predicts between 0% and 200% liver loss over the first 100 days in bi-phasic V(t) patterns (see Figure 6A, blue shade). By contrast, model (1) predicts between 150% and 300% liver loss in the tri-phasic V(t) samples (see Figure 6A, pink shade). In both patterns, the loss is compensated by cell division. In particular, for the $r_{T}/r_{I}=5$, the net liver gain leads to an increase in the overall liver size up to 50% for both V(t) patterns (see Figure 6B). There is little difference between the net liver gain over the first 100 days in the bi-phasic and tri-phasic V(t) patterns (see Figure 6B, blue versus pink shades). If we increase drug efficacy, then the liver turnover and net liver gain decrease for both bi-phasic and tri-phasic samples, even when decreasing the $r_{T}/r_{I}$ ratio (see Figure S3). Indeed, for ϵ=0.99 and $r_{T}/r_{I}=2.5$, we predict between 0% and 70% liver loss over the first 100 days in bi-phasic V(t) patterns (see Figure S3A, blue shade); and between 25% and 300% liver loss in the tri-phasic V(t) samples (see Figure S3A, pink shade).

**Figure 7:**
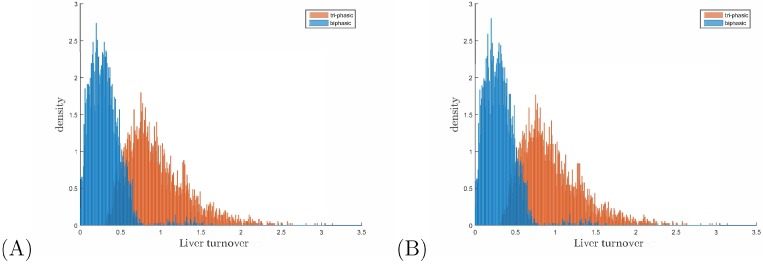
Density of bi-phasic (blue) and tri-phasic (pink) V(t) samples versus liver turnover for V(t) curves that achieved clearance (of one virion) in (**A**) one year; (**B**) two years. The parameters are fixed ϵ=0.99 ad $r_{T}/r_{I}=2.5$, and variable 0.01≤δ≤0.1 $d^{-1}$.

Experimental and modeling studies have estimated the liver turnover when the infection is resolved to range between 69% and 100% [14,27,28,29]. We looked at all the samples that lead to virus clearance in the first year and in the second year for fixed drug efficacy ϵ=0.99, fixed $r_{T}/r_{I}=2.5$ and varying hepatocyte killing rates 0.01≤δ≤0.1. We found that the hepatocyte turnover at the time of virus clearance is, on average, 0.3 for the bi-phasic samples and 0.95 for the tri-phasic samples for both years (see Figure 7 for the full density plots).

**Figure 8:**
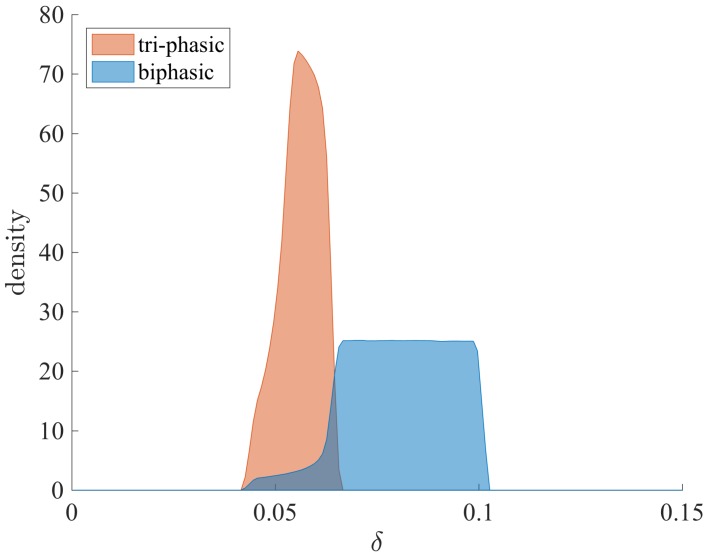
Density of bi-phasic (blue) and tri-phasic (pink) V(t) samples versus δ, for fixed ϵ=0.7 and $r_{T}/r_{I}=5$.

The prediction that the samples explaining tri-phasic V(t) patterns show higher liver turnover over the first 100 days is in conflict with the fact that such samples favor low or similar values for the cytotoxic killing rate δ (Figure 8). Since this elevated liver loss cannot be explained by high δ values, it has to be explained by high I(t) levels at the start of the therapy in all tri-phasic samples. That means, the samples corresponding to tri-phasic V(t) patterns are also samples that describe an infection with large concentration of cells being infected at the beginning of treatment, compared to the samples corresponding to bi-phasic V(t) patterns.

**Figure 9:**
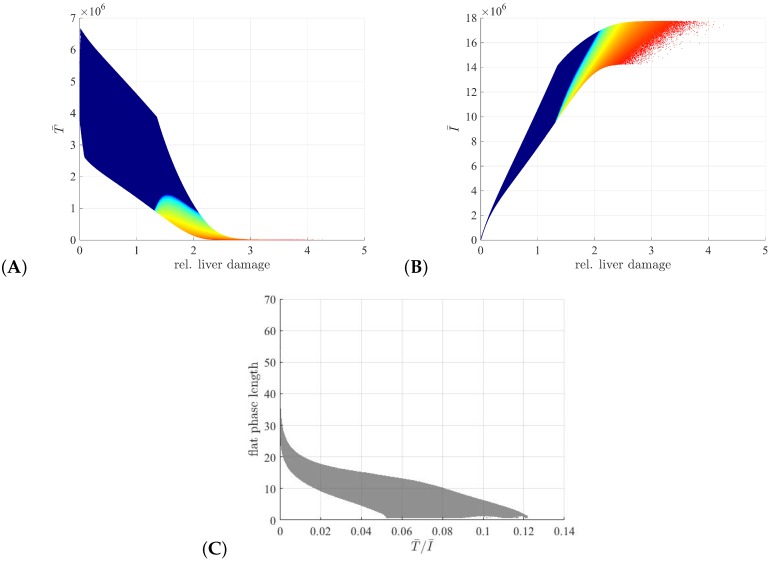
Samples for bi-phasic (dark blue dots) and tri-phasic (red to light blue dots) V(t) dynamics for ϵ=0.7, $r_{T}/r_{I}=5$, 0.01≤δ≤0.1 $d^{-1}$, τ=100 days in respect to: (**A**) liver turnover and initial uninfected liver population $\bar{T}$, (**B**) liver turnover and initial uninfected liver population $\bar{T}$. (**C**) Length of the third phase as a function of the initial conditions $\bar{T}/\bar{I}$.

To determine the relationship between the initial conditions and the V(t) decay pattern, we plot the initial conditions for uninfected and infected hepatocyes at the start of the treatment, $\bar{T}$ and $\bar{I}$ as given by (2), over the hepatocyte turnover for each sample generated in the subcase S4: case $r_{T}/r_{I}=5$, ϵ=0.7 and 0.01≤δ≤0.1 $d^{-1}$. We observe that the uninfected liver population is low while the infected liver population is high (with at least a 10% to 90% ratio) in all tri-phasic samples (Figure 9A,B, red-to-ligt blue dots). By contrast, in the bi-phasic samples the uninfected population dominates the overall liver population (Figure 9A,B, dark blue dots). Most importantly, this is the only result, where there is no overlap between parameter samples giving the bi-phasic and tri-phasic V(t) patterns. Moreover, the length of flat phase in the tri-phasic samples correlates with the uninfected to infected cell ratio $\bar{T}/\bar{I}$ at the beginning of treatment (Figure 9C). This suggests that the size of the infection is important in determining the type of virus decay following initiation of therapy, with tri-phasic V(t) patterns with long flat phases occurring in the cases where the majority of the liver is infected.

## 4. Discussion

In this study, we investigate the mechanisms of virus decay in hepatitis B infections following the initiation of drug therapy. Data from drug clinical studies have shown that HBV DNA follows either a bi-phasic or a tri-phasic decay pattern [11,16,17,18]. Modeling studies have defined the two stages of bi-phasic virus decay to represent a rapid initial removal of free virus followed by a slower removal of infected cells [10,11,21,30]. Less is known regarding the mechanisms behind the emergence of the flat phase in the tri-phasic viral decay pattern. It may be due to pharmacological and/or virological reasons, such as emergence of drug-resistant strains.

Using the mathematical model developed in [12], we show that immunological, virological and pharmacological factors do contribute to the patterns followed by the virus population. Our study considers the following factors: (1) drug efficacy; (2) therapy duration; (3) ratio between division of uninfected and infected cells; (4) strength of cytotoxic immune response; (5) level of liver infection at the beginning of the treatment and (6) net liver gain.

To determine the relative contributions of these parameters and mechanisms we employ a statistical approach. By using rejection sampling methods we eliminate the parameter samples which do not lead to feasible virus dynamics. The distribution of the remaining parameters reveal how the virus dynamics are influenced by the sizes and correlation of parameters.

We find that high drug efficacy is needed for any type of virus decay. Moreover, high uninfected to infected division rate combined with high immune response induced cytotoxic killing of infected cells result in bi-phasic viral decay patterns. This result indicates that bi-phasic decay patterns are better for a patient prognosis. We show that activation of the immune responses following the start of therapy is beneficial for the patient prognosis. This finding is supported by other experimental [31,32] and modeling studies [33].

For therapy that is 99% efficacious, we found that successful and partially successful therapies, which lead to virus clearance in the first and second year of treatment, are accompanied by 30% and 95% liver turnover for the bi-phasic and tri-phasic cases. The results are similar to previous modeling and experimental measurements, which estimated the liver turnover at the virus resolution to range between 69% and 100% [14,27,28,29].

Our investigations could not find significant differences between the pattern of virus decay when either the uninfected to infected cell division rate, the immune response killing rate, or both have small or intermediate values. For such ranges, both bi-phasic and tri-phasic viral decay patterns can be observed. Moreover, for these small to intermediate $r_{T}/r_{I}$ and δ values, the amount of time it takes to reach virus clearance (described as less than on virion in the body) is independent of the pattern of decay.

On the other hand, in the tri-phasic samples, we find a correlation between high level of hepatocyte infection at the start of the treatment (greater than 85%) and increased liver turnover. High levels of liver infection have been reported in acute HBV infections in chimpanzees and woodchucks [15]. The exact levels of HBV infections in chronic human infections are considered to be much lower, although the exact infection level is rarely documented. Do patients experiencing higher levels of hepatocyte infection have higher base virus levels at the start of the treatment? Our model assumes that the virus is in quasi-equilibrium with the infected hepatocytes at the start of the infection, and, consequently, high base virus concentrations are attained for all tri-phasic cases.

Our model does not consider non-cytotoxic immune responses, nor did it consider the relation between the liver resolution and the patient outcome. Data on the loss of other virological markers, such as the covalently closed circular DNA and surface antigens, are needed to further assess the relationship between the pattern of virus decay and the success of treatment.

In conclusion, we have investigated the mechanisms behind two types of hepatitis B virus decay patterns following the start of drug therapy and found that high uninfected to infected division rate combined with high cytotoxic killing of infected cells results in bi-phasic virus decay, while infection of a large percentage of liver at the beginning of treatment results in tri-phasic virus decay.

## Figures and Tables

**Table 1:** Parameter values (see Table 2 for definitions) for the different viral decay profiles in Figure 1.

Figure 1	β	$r_{T}$	$r_{T}/r_{I}$	*K*	δ	*p*	*c*	η	ϵ
(A)	$1\cdot 10^{-10}$	3	5	$1.9\cdot 10^{7}$	0.07	100	0.67	0.5	0.99934
(B)	$2.5\cdot 10^{-7}$	0.9	3.3	$1.9\cdot 10^{7}$	0.22	5	0.7	0.5	0.991
(C)	$1.9\cdot 10^{-6}$	0.8	2.9	$1.9\cdot 10^{7}$	0.25	1.4	0.18	0.2	0.9988
(D)	$6.6\cdot 10^{-8}$	0.5	7.2	$1.9\cdot 10^{7}$	0.06	164	1	0.5	0.997

**Table 2:** Fixed and boundaries for the parameters in model (1).

Parameter	Description	Value	Reference
β	Infectivity rate	$10^{-10}$ mL/(virions × d)	[22]
*K*	Hepatocyte-carrying capacity	$1.9\times 10^{7}$ cells/mL	[12]
*p*	Viral production	100 virions/(cell × d)	[22]
*c*	Viral clearance	0.67 $d^{-1}$	[21]
η	Efficacy of therapy in blocking infection	0.5	[10]
**Parameter**	**Description**	**Range**	**References**
$r_{T}$	Uninfected cell division rate	(0,4] $d^{-1}$	[12,22]
$r_{I}$	Infected cell division rate	(0,4] $d^{-1}$	[12]
δ	Infected cells killing rate	[0.01,0.1] $d^{-1}$	[10,11,12,22]
ϵ	Efficacy of therapy in blocking viral production	[0.2,1]	[10,12]

## Supplementary Materials

The supplementary materials are available online at www.mdpi.com/1999-4915/9/5/117/s1.

## References

[1] Locarnini S., Hatzakis A., Chen D.S., Lok A. (2015). Strategies to control hepatitis B: Public policy, epidemiology, vaccine and drugs. J. Hepatol..

[2] Chen D.S. (2009). Hepatitis B vaccination: The key towards elimination and eradication of hepatitis B. J. Hepatol..

[3] Manzoor S., Saalim M., Imran M., Resham S., Ashraf J. (2015). Hepatitis B virus therapy: What’s the future holding for us?. World J. Gastroenterol..

[4] World Health Organization (2016). Hepatitis B Fact sheet 204.

[5] Lampertico P., Aghemo A., Viganò M., Colombo M. (2009). HBV and HCV therapy. Viruses.

[6] Sonneveld M., Wong V., Woltman A., Wong G., Cakaloglu Y., Zeuzem S., Buster E., Uitterlinden A., Hansen B., Chan H. (2012). Polymorphisms Near IL28B and Serologic Response to Peginterferon in HBeAg-Positive Patients With Chronic Hepatitis B. Gastroenterology.

[7] Hadziyannis S., Tassopoulos N., Heathcote E., Chang T.T., Kitis G., Rizzetto M., Marcellin P., Lim S., Goodman Z., Wulfsohn M. (2003). Adefovir Dipivoxil for the Treatment of Hepatitis B e Antigen–Negative Chronic Hepatitis B. N. Engl. J. Med..

[8] Chan H., Wang H., Niu J., Chim A., Sung J. (2007). Two-year lamivudine treatment for hepatitis B e antigen-negative chronic hepatitis B: A double-blind, placebo-controlled trial. Antivir. Ther..

[9] Lok A., McMahon B., Brown R., Wong J., Ahmed A., Farah W., Almasri J., Alahdab F., Benkhadra K., Mouchli M. (2016). Antiviral therapy for chronic hepatitis B viral infection in adults: A systematic review and meta-analysis. Hepatology.

[10] Lewin S.R., Ribeiro R.M., Walters T., Lau G.K., Bowden S., Locarnini S., Perelson A.S. (2001). Analysis of hepatitis B viral load decline under potent therapy: complex decay profiles observed. Hepatology.

[11] Tsiang M., Rooney J.F., Toole J.J., Gibbs C.S. (1999). Biphasic clearance kinetics of hepatitis B virus from patients during adefovir dipivoxil therapy. Hepatology.

[12] Dahari H., Shudo E., Ribeiro R.M., Perelson A.S. (2009). Modeling complex decay profiles of Hepatitis B virus during antiviral therapy. Hepatology.

[13] Seeger C., Mason W. (2016). HBV replication, pathobiology and therapy: Unanswered questions. J. Hepatol..

[14] Murray J., Goyal A. (2015). In silico single cell dynamics of hepatitis B virus infection and clearance. J. Theor. Biol..

[15] Guidotti L., Rochford R., Chung J., Shapiro M., Purcell R., Chisari F. (1999). Viral clearance without destruction of infected cells during acute HBV infection. Science.

[16] Ribeiro R., Germanidis G., Powers K., Pellegrin B., Nikolaidis P., Perelson A., Pawlotsky J.M. (2010). Hepatitis B Virus Kinetics under Antiviral Therapy Sheds Light on Differences in Hepatitis B e Antigen Positive and Negative Infections. J. Inf. Dis..

[17] Lau G., Tsiang M., Hou J., Yuen S., Carman W., Zhang L., Gibbs C. (2000). Combination therapy with lamivudine and famciclovir for chronic hepatitis B-infected Chinese patients: A viral dynamics study. Hepatology.

[18] Colombatto P., Civitano L., Bizzarri R., Oliveri F., Choudhury S., Gieschke R. (2006). A multiphase model of the dynamics of HBV infection in HBeAg-negative patients during pegylated interferon-alpha2a, lamivudine and combination therapy. Antivir. Ther..

[19] Kim H., Kwon H.D., Jang T., Lim J., Lee H.S. (2012). Mathematical Modeling of Triphasic Viral Dynamics in Patients with HBeAg-Positive Chronic Hepatitis B Showing Response to 24-Week Clevudine Therapy. PLoS ONE.

[20] Ciupe S., Ribeiro R., Nelson P., Perelson A. (2007). Modeling the mechanisms of acute hepatitis B virus infection. J. Theor. Biol..

[21] Nowak M.A., Bonhoeffer S., Hill A.M., Boehme R., Thomas H.C., McDade H. (1996). Viral dynamics in Hepatitis B virus infection. Proc. Natl. Acad. Sci. USA.

[22] Ciupe S., Ribeiro R., Nelson P., Dusheiko G., Perelson A. (2007). The role of cells refractory to productive infection in acute hepatitis B viral dynamics. Proc. Natl. Acad. Sci. USA.

[23] Haussinger D. (2011). Liver Regeneration.

[24] Robert C., Casella G. (2013). Monte Carlo Statistical Methods.

[25] Chisari L.G.F. (2006). Immunobiology and pathogenesis of viral hepatitis. Annu. Rev. Pathol..

[26] van Thiel D.H., Gavaler J.S., Kam I., Francavilla A., Polimeno L., Schade R.R., Smith J., Diven W., Penkrot R.J., Starzl T.E. (1987). Rapid Growth of an Intact Human Liver Transplanted Into a Recipient Larger Than the Donor. Gastroenterology.

[27] Summers J., Jilbert A., Yang W., Aldrich C., Saputelli J., Litwin S., Toll E., Mason W. (2003). Hepatocyte turnover during resolution of a transient hepadnaviral infection. Proc. Natl. Acad. Sci. USA.

[28] Mason W., Litwin S., Xu C., Jilbert A. (2007). Hepatocyte turnover in transient and chronic hepadnavirus infections. J. Viral Hepat..

[29] Mason W., Xu C., Low H., Saputelli J., Aldrich C., Scougall C., Grosse A., Colonno R., Litwin S., Jilbert A. (2009). The amount of hepatocyte turnover that occurred during resolution of transient hepadnavirus infections was lower when virus replication was inhibited with entecavir. J. Virol..

[30] Zeuzem S., de Man R., Honkoop P., Roth W., Schalm S., Schmidt J. (1997). Dynamics of hepatitis B virus infection in vivo. J. Hepatol..

[31] Chen Y., Chu C., Liaw Y. (2010). Age-Specific Prognosis Following Spontaneous Hepatitis B e Antigen Seroconversion in Chronic Hepatitis B. Hepatology.

[32] Brunetto M., Giarin M., Oliveri F., Chiaberge E., Baldi M., Alfarano A., Serra A., Saracco G., Verme G., Will H. (1991). Wild-type and e antigen-minus hepatitis B viruses and course of chronic hepatitis. Proc. Natl. Acad. Sci. USA.

[33] Ciupe S., Hews S. (2012). Mathematical models of e-antigen mediated immune tolerance and activation following prenatal HBV infection. PLoS ONE.

