# Supplementary material for: Understanding the Complex Patterns Observed during Hepatitis B Virus Therapy
Source: Viruses. 2017 May 19;9(5):117. doi: 10.3390/v9050117 (PMC5454429; doi:10.3390/v9050117)
Supplement: Supplementary file 1 [file viruses-09-00117-s001.pdf]

Understanding the complex patterns observed during hepatitis B Virus therapy - Supplementary material

Andrea Carracedo Rodriguez*, **Matthias Chung***, **Stanca M Ciupe***,¶

April 21, 2017

Positivity and boundness. We first establish the positivity and boundness of model

$$\begin{aligned} \frac{\mathrm{d}\,T}{\mathrm{d}\,t} &= r_T T\left(1-\frac{T+I}{K}\right) - \beta(1-\eta)VT, \\ \frac{\mathrm{d}\,I}{\mathrm{d}\,t} &= r_I I\left(1-\frac{T+I}{K}\right) + \beta(1-\eta)VT - \delta I, \\ \frac{\mathrm{d}\,V}{\mathrm{d}\,t} &= (1-\epsilon)pI - cV. \end{aligned} \tag{1}$$

subject to initial conditions

$$T(0) = T_0 > 0, \qquad I(0) = I_0 > 0, \qquad V(0) = V_0 > 0, \qquad T(0) + I(0) \leq K. \tag{2}$$

Proposition 1. *The solutions of* (1) *subject to* (2) *are positive on* $[0, b)$ *for some* $b > 0$.

Proof. Note that (1) is locally Lipschitz at $t = 0$. Therefore, a solution exists and is unique on $[0, b)$ for some $b > 0$. Assume that there exists $t_1 \in (0, b)$ such that $V(t_1) = 0$ and all variables are positive on $[0, t_1)$. For all $t \in [0, t_1]$

$$\frac{\mathrm{d}\,V}{\mathrm{d}\,t} = (1-\epsilon)pI - cV \geq -cV,$$

and so

$$V(t_1) \geq V_0 e^{-ct_1} > 0,$$

a contradiction. Therefore $V(t) > 0$ for all $t \in [0, t_1]$.

□

Proposition 2. *If* $\max\{r_T, r_I\} > \min\{d_T, \delta\}$, *then any solution* $(T(t), I(t))$ *of* (1) *subject to* (2) *remains bounded on* $[0, b)$ *for some* $b > 0$.

Proof. Let $F = T + I$, $r_{\max} = \max\{r_T, r_I\}$ and $d_{\min} = \min\{d_T, \delta\}$. Then

$$\begin{aligned} \frac{dF}{dt} &\leq s + r_{\max}F\left(1-\frac{F}{K}\right) - d_{\min}F \\ &= s + (r_{\max} - d_{\min})F - \frac{r_{\max}}{K}F^2 \\ &= -\frac{r_{\max}}{K}(F-X)(F-Y), \end{aligned}$$

where

¶Corresponding author

$$X = \frac{r_{\max} - d_{\min} + \sqrt{(r_{\max} - d_{\min})^2 + \dfrac{4sr_{\max}}{K}}}{-2s},$$

$$Y = \frac{r_{\max} - d_{\min} - \sqrt{(r_{\max} - d_{\min})^2 + \dfrac{4sr_{\max}}{K}}}{-2s}.$$

Then

$$\int \frac{dF}{(F-X)(F-Y)} \leq \int -\frac{r_{\max}}{K} dt$$

$$\frac{1}{X-Y} \ln|F-X| + \frac{1}{Y-X} \ln|F-Y| \leq -\frac{r_{\max}}{K} t$$

$$\left|\frac{F-X}{F-Y}\right| \leq \exp\left(-\frac{(X-Y)r_{\max}}{K} t\right).$$

and, since $r_{\max} > d_{\min}$,

$$F(t) \leq \frac{X - Y \exp\left(-\frac{(X-Y)r_{\max}}{K} t\right)}{1 - \exp\left(-\frac{(X-Y)r_{\max}}{K} t\right)}.$$

Note that $X - Y > 0$, and so $F(t)$ is bounded. Therefore $T(t)$ and $I(t)$ are bounded.

□

Proposition 3. *Any solution $(T(t), I(t))$ of (1) subject to (2) is positive on $[0, b)$ for some $b > 0$.*

Proof. We first show positivity for F. Assume that there exists $t_1 \in (0, b)$ such that $F(t_1) = 0$ and all variables are positive on $[0, t_1)$. Assume also that T and I are bounded on $[0, t_1)$, i.e., there exist M_1 and M_2 such that $T(t) \leq M_1$ and $I(t) \leq M_2$ for all $t \in [0, t_1)$. Then for all $t \in [0, t_1]$

$$\begin{aligned}
\frac{\mathrm{d}F}{\mathrm{d}t} &\geq r_{\min} F\left(1 - \frac{F}{K}\right) - d_{\max} F \\
&\geq r_{\min} F\left(-\frac{M_1 + M_2}{K}\right) - d_{\max} F \\
&= -\tilde{c} F, \qquad\qquad \tilde{c} > 0
\end{aligned}$$

and so

$$F(t_1) \geq F_0 e^{-\tilde{c} t_1} > 0$$

a contradiction. Then $F(t) > 0$ for all $t \in [0, t_1]$. Since we assume all the variables positive on $[0, t_1)$, this implies that both $T(t)$ and $I(t)$ are positive for all $t \in [0, t_1]$.

□

Proposition 4. *If* $\max\{r_T, r_I\} > \min\{d_T, \delta\}$, *then any solution* $V(t)$ *of* (1) *subject to* (2) *remains bounded on* $[0, b)$ *for some* $b > 0$.

Proof. If $I(t)$ is bounded on $[0, b)$, then there exists a number $M > 0$ such that

$$M \geq (1-\epsilon)p \sup_{t\in[0,b)} I(t).$$

Then for any $t \in [0, b)$ we have

$$\frac{\mathrm{d}V}{\mathrm{d}t} = (1-\epsilon)p - cV \leq M - cV,$$

and so

$$V(t) \leq \max\left\{V_0, \frac{M}{c}\right\}.$$

□

Stability analysis. We study the local asymptotic stability of system (1)'s equilibria for $\epsilon = \eta = 0$. The system has four equilibria: a liver death equilibria $E^* = (0, 0, 0)$, a disease-free equilibrium

$$E_0 = (K, 0, 0),$$

a chronic infection equilibrium with total liver infection

$$E^{tot.liv} = \left(0, \frac{K(r_I - \delta)}{r_I}, \frac{pK(r_I - \delta)}{cr_I}\right),$$

and a chronic equilibrium with partial liver infection

$$E = \left(\frac{c\delta(R_0(\delta - r_I) + r_T)}{\beta p(R_0\delta + r_T - r_I)}, \frac{c\delta r_T(R_0 - 1)}{\beta p(R_0\delta + r_T - r_I)}, \frac{\delta r_T(R_0 - 1)}{\beta(R_0\delta + r_T - r_I)}\right),$$

where

$$R_0 = \frac{\beta pK}{c\delta} \tag{3}$$

is the basic reproduction number, representing the number of secondary infections induced by an infected cell in a naive population.

Proposition 5. *The liver death equilibrium is unstable.*

Proof. The Jacobian matrix for the system is

$$J = \begin{pmatrix} r_T\left(1 - \frac{2T+I}{K}\right) - \beta V & -r_T\frac{T}{K} & -\beta T \\ -r_I\frac{I}{K} + \beta V & r_I\left(1 - \frac{T+2I}{K}\right) - \delta & \beta T \\ 0 & p & -c \end{pmatrix}. \tag{4}$$

When evaluated at E^*, J becomes:

$$J = \begin{pmatrix} r_T & 0 & 0 \\ 0 & r_I - \delta & 0 \\ 0 & p & -c \end{pmatrix},$$

whose eigenvalues $\lambda_1 = r_T > 0$ and $\lambda_2 = r_I - \delta > 0$. Therefore E^* is unstable.

□

Proposition 6. *The free disease equilibrium is locally asymptotically stable if* $R_0 < 1$.

Proof. The Jacobian matrix for the system evaluated at E_0 becomes:

$$J = \begin{pmatrix} -r_T & -r_T & -\beta K \\ 0 & -\delta & \beta K \\ 0 & p & -c \end{pmatrix},$$

whose eigenvalues are negative when $R_0 < 1$.

□

Proposition 7. *The equilibrium* $E^{tot.liv}$ *exists when* $r_I > \delta$, *is locally asymptotically stable when*

$$R_0 \frac{r_I - \delta}{r_T} > 1,$$

and is unstable otherwise.

Proof. $E^{tot.liv}$ exists when $r_I > \delta$. It can be shown that the characteristic equation for $E^{tot.liv}$ is given by

$$(\lambda - \frac{r_T\delta}{r_I} + \frac{\beta p K(r_I - \delta)}{r_I c})(\lambda + c)(\lambda + r_I - \delta) = 0,$$

with eigenvalue $\lambda_1 = \frac{r_T\delta}{r_I} - \frac{\beta p K(r_I-\delta)}{r_I c} < 0$ when $R_0\frac{r_I-\delta}{r_T} > 1$. Since the other two eigenvalues are always negative, this condition is enough to ensure local asymptotic stability of equilibrium $E^{tot.liv}$.

□

Proposition 8. *The equilibrium* E *is locally asymptotically stable if* $r_I > \delta$ *and*

$$1 < R_0 \text{ and } R_0\frac{r_I - \delta}{r_T} < 1.$$

The proof is messy and it will not be presented here.

When the treatment is initiated, we assume that the chronic equilibrium E is stable, *i.e.* $1 < R_0 < \frac{r_T}{r_I-\delta}$. A successful combination drug therapy $0 < \epsilon \leq 1$ and $0 < \eta \leq 1$ will lead to virus clearance if the clearance equilibrium in the presence of therapy, $E_0^d = (K, 0, 0)$ (same as E_0 in the absence of therapy), becomes the locally asymptotically stable steady state. This occurs when

$$\mathcal{R} = (1 - \epsilon)(1 - \eta)R_0 < 1. \tag{5}$$

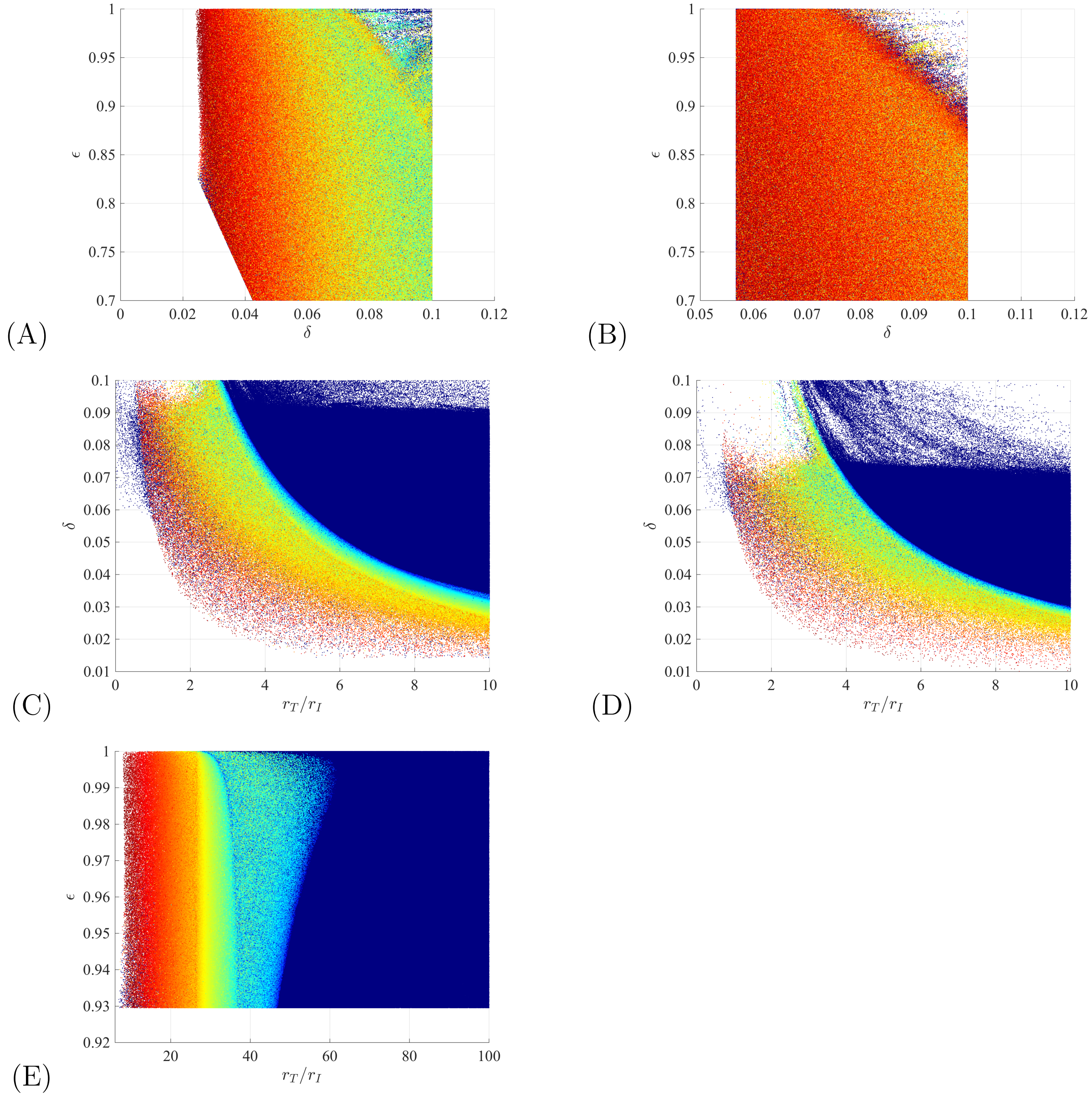

Figure S1: Samples for bi-phasic (dark blue dots) and tri-phasic (red to light blue dots) $V(t)$ dynamics for: (A) fixed $r_T/r_I = 2.5$; (B) fixed $r_T/r_I = 1$; (C) fixed $\epsilon = 0.9$; and (D) fixed $\epsilon = 0.99$; (E) fixed $\delta = 0.01$. The other parameters are as in Table 2.

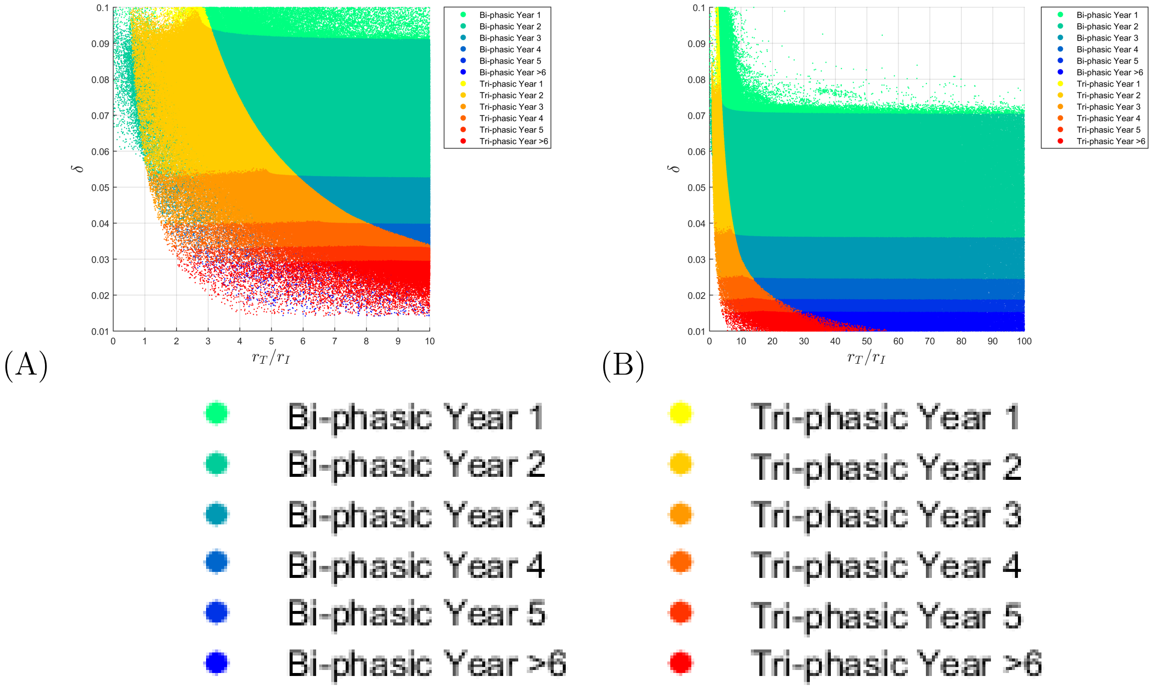

Figure S2: Division of the samples for bi-phasic and tri-phasic virus pattern based on the number of years to virus clearance for: (A) fixed $\epsilon = 0.9$; (B) fixed $\epsilon = 0.99$. The other parameters are as in Table 2.

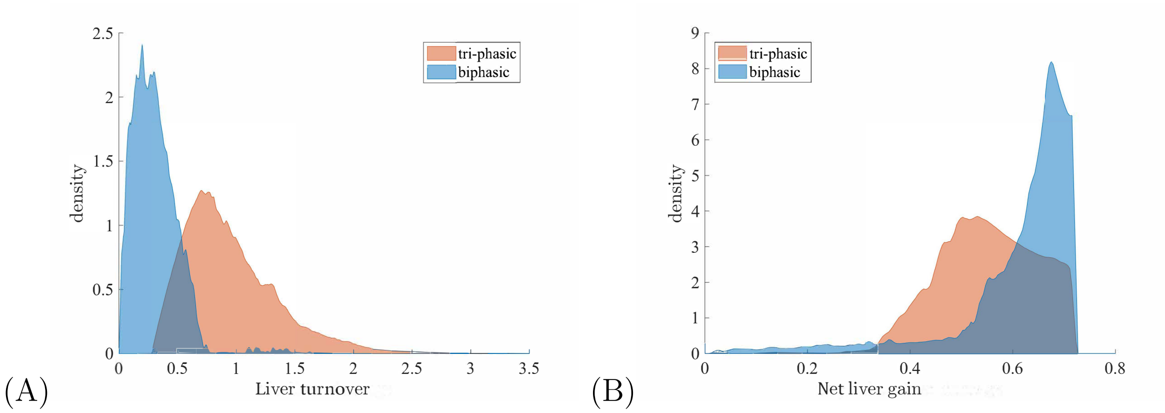

Figure S3: Density of bi-phasic (blue) and tri-phasic (pink) $V(t)$ samples versus: (A) Liver turnover; (B) Net liver gain, for $\epsilon = 0.99$, $r_T/r_I = 2.5$, $0.01 \leq \delta \leq 0.1$ d^{-1} and $\tau = 100$ days.
